# Comparing Facial Emotional Recognition in Patients with Borderline Personality Disorder and Patients with Schizotypal Personality Disorder with a Normal Group

**Published:** 2017-04

**Authors:** Aida Farsham, Tahereh Abbaslou, Reza Bidaki, Bonnie Bozorg

**Affiliations:** 1 Clinical Psychologist, Alzahra University, Tehran, Iran.; 2 Clinical Psychologist, Islamic Azad University of Semnan, Semnan, Iran.; 3 Psychiatrist, Associate Professor, Research Center of Diabetes, Shahid Sadoughi University of Medical Sciences, Yazd, Iran.; 4 Clinical Psychologist, Tehran University of Medical Sciences, Tehran, Iran.

**Keywords:** *Borderline Personality Disorder*, *Schizotypal Personality Disorder*, *Facial Emotional Recognition*

## Abstract

**Objective:** No research has been conducted on facial emotional recognition on patients with borderline personality disorder (BPD) and schizotypal personality disorder (SPD). The present study aimed at comparing facial emotion recognition in these patients with the general population. The neurocognitive processing of emotions can show the pathologic style of these 2 disorders.

**Method: **Twenty BPD patients, 16 SPD patients, and 20 healthy individuals were selected by available sampling method. Structural Clinical Interview for Axis II, Millon Personality Inventory, Beck Depression Inventory and Facial Emotional Recognition Test was were conducted for all participants.

**Discussion: **The results of one way ANOVA and Scheffe’s post hoc test analysis revealed significant differences in neuropsychology assessment of facial emotional recognition between BPD and SPD patients with normal group (p = 0/001). A significant difference was found in emotion recognition of fear between the 2 groups of BPD and normal population (p = 0/008). A significant difference was observed between SPD patients and control group in emotion recognition of wonder (p = 0/04(.

The obtained results indicated a deficit in negative emotion recognition, especially disgust emotion, thus, it can be concluded that these patients have the same neurocognitive profile in the emotion domain.

Personality disorder is a common and chronic disorder in 10% to 15% of the general population (1). Biomedical and psychological aspects of personality disorders are quite complicated. There is some similarity between schizotypal (cluster A personality disorder) and borderline personality disorder that are caused by stressor. Both of them are placed in a border of psychosis situation (2). Krenberg believe that some personality disorders in Diagnostic and Statistical Manual of Mental Disorder (DSM) classification have been described in borderline personality disorder (BPD). A basic 

Difference between neurosis and borderline personality structure is the possibility of impairment in reality testing at the borderline personality. Patients with SPD may also show some features of borderline personality disorder and both disorders can be diagnosed at the same time. These two personality disorders are similar in some aspects such as inability to establish close relations, inappropriate affect, or restricted and suspicious, or paranoid thoughts. Patients with borderline personality disorder and schizotypal personality disorders (SPD) are poorer in psychosocial functions than other disorders (3). Rawlings et al.

reported a significant correlation between schizotypal and borderline traits (4). Previous studies have demonstrated that borderline patients have more problems in structure, discrimination, and integration of emotions, and react more intensely to negative emotional stimuli compared to control group (5). Functional magnetic resonance imaging (fMRI) studies have shown that borderline personality disorder symptoms are related to Fronto-limbic circuits (6). Among subcortical limbic structures, amygdala plays an important role in social and emotional processes (7). Consequently, patients with BPD over respond to emotional and social stimuli (8), (9).

Donegan et al. observed amygdala activity more than they expected in 15 patients with BPD when the patients saw neutral, happy, sad, or fearful faces. Patient with borderline personality disorder interpreted neutral faces as worthless, threatening, and negative. Nevertheless, all patients with BPD do not show deficit of emotional processing (10). Wagner and Linehan compared emotion recognition in 21 females with BPD, 21 females with a history of sexual abuse without BPD, and 20 healthy controls. Results indicated that patients with BPD perceive others emotion carefully, especially fears. No significant difference was observed in facial emotional recognition among the 3 groups (11). There are few studies about SPD’s cognitive function due to the clinical definition of this disorder. Poureh et al. found that patients with schizotypal features are worse in identifying facial emotional than control group. Williams et al. showed that the severity of schizotypal trait is associated with weakness of emotional recognition, especially positive emotion. Some studies assume that emotional recognition deficit is because of disability in discrimination of others’ emotions (12). Research displays that patients with SPD show a negative bias for vague or neutral faces. In addition, schizophrenic patients with hallucination have attention bias for nervous facial expressions (13). Also, Brown and Cohen found that patients with SPD misrecognize neutral faces as disgust in comparison to control group. Moreover, patients with SPD and control group had no difference in reaction time during detecting facial emotion. Comparing these 2 groups of mental disorders is difficult and complex because there are some similarities between classifications of mental disorders. Neuropsychological tests are useful to assess the differentiation of symptoms (14).

The present research raise the following question: Are there any differences between patients with BPD and patients with SPD in facial emotion recognition?

## Materials and Methods

This was a cross sectional ex post facto sectional survey. Facial emotion recognition was studied among patients with diagnosis of borderline personality disorder and schizotypal personality disorder according to DSM -IV -TR and normal population. 

***Participants***

The study population included all BPD and SPD patients referred to Imam Hussein hospital (AS), Imam Khomeini hospital, and Isatis psychological clinic in North of Tehran. A nonrandomized available sampling was done because of time limitation and loss of patients. Three groups of patients with borderline personality disorder, schizotypal personality disorder, and normal population participated in this study.

The sample consisted of 56 individual (20 BPD patients, 16 SPD, and 20 samples of normal population). The inclusion criteria for normal group were absence history of mental illness. Demographic variables such as age, education, marital and employment status were matched in the 3 groups.

***Procedure***

At first, psychiatry interview was done to make rapport between the patients and the researcher; this interview had led to psychiatric diagnosis. Finally, structural Clinical Interview for Axis II was used to confirm the diagnosis. The Millon Personality Inventory and Beck Depression Inventory were used to screen the normal group. After describing the aims of the study to the participants, informed consent was obtained from each of them. Eventually, facial emotion recognition test was accomplished. Rest time was considered during the interview and test to promote efficiency.

***Instrument***

1.           Structured Clinical Interview for Axis II, DSM-IV: SCID-II is a semi-structure diagnostic interview used to assess 10 personality disorders patients according to DSM-IV Axis II (First and Gibbons). Disorders placed in axis II can be recognized into 2 forms: having classification, or dimension (according to the criteria for personality disorder).

2.           Millon Clinical Multiaxial Inventory III (MCMI- III, Millon 1994): A self-report Inventory includes 175 yes or no items that assesses 14 clinical patterns of personality and 10 clinical syndromes. It can be used for psychological assessment and treatment of adults above 18 years who had been referred to mental health centers. This test is one of the most important measures for assessing objective clinical syndromes in DSM-IV Axis I and Axis II personality disorders. MCMI III tries to predict existence or absence of clinical disorders by using baseline scores. Cut-off point of this inventory is 85.

3.           Beck Depression Inventory: The questionnaire is a multiple choice self-report with 21 questions that measuring the severity of depression. It is based on Likert scoring (0 to 3). The score range of the inventory is 0 to 63.

4.           Facial Emotion Recognition Test: In the present study, the participants recognized emotions by 60 images out of 110 images (the 6 basic emotions of 10). Audiovisual software with ability of playback and recording answers was used to run tests and collect data. Images were shown to the participants. Six basic emotions were indicated with 6 marked keys on the keyboards. Then, participants identified each of the 6 basic emotions by pressing the related keys. Each image was shown on the monitor between 400 and 500 milliseconds (15); 900 MS was the interval between 2 images. The test lasted almost 2 minutes. The number of correct responses, incorrect, and no answer (missed answer) was recorded by the software. In this study, the test images were based on one of the most common and reliable tests According to research by Ekman, these 60 images have good reliability and validity. The rate of correct answers to these 60 images was 91% (16). 

## Results

Twenty cases participated in the present study as BPD patients (6 males and 14 females), 16 SPD patients (6 males and 10 females) and 20 individuals (9 males and 11 females,) as a control group. A large number of participants were single aged 20 to 30 years. Most of them had a bachelor’s degree in all groups. The results of the ANOVA test did not reveal significant differences among the 3 groups in depression variable. Depression did not have an effect on the neurological function differences of these 3 groups. The average number of incorrect answers was higher in SPD compared to BPD groups compared to the control group. The control group had the highest average in correct responses among the 3 groups. 

The results of the ANOVA revealed a significant difference in fear, happiness, hate, and surprise emotions in the total number of correct responses among the 3 groups. Moreover, significant differences were obtained in the total number of incorrect responses of 3 groups in hate and surprise emotions. Scheffe’s post hoc test was used to specify the exact difference among groups. The results are presented in the following table: According to Table 2, a significant difference was observed in the total correct and incorrect answers between BPD and normal group and SPD and normal group. Moreover, a significant difference was obtained in correct responses about fear and happiness emotions between BPD and normal group. Also, meaningful differences were observed in the correct and incorrect responses in surprise emotions between SPD and normal group. In addition, a significant difference was detected in hate emotion for the correct and incorrect responses between SPD and BPD groups and SPD and normal group. 

**Table1:** ANOVA Test Results for Facial Emotion Recognition

**VARIABLE**		**DF**	**SS**	**MS**	**F**
**Total correct** **responses**	Between group	2	561.879	280.939	6.320*
	Within group	53	2356.050	44.454	
	total	55	2917.929		
**Total incorrect** **responses**	between group	2	408.112	204.056	8.169**
	Within group	53	1323.888	24.979	
	Total	55	1732.000		
**Total non-** **responses**	Between group	2	21.041	10.521	0.595
	Within group	53	936.888	17.677	
	Total	55	957.929		

* P<0/01,

** P<0/001

**Table2:** Scheffe’s Post Hoc Test Results in Facial Emotion Recognition

**Variable**	**Group**	**Group**	**V**	**SD**	**Sig**
**Total correct ** **responses**	BPD	SPD	1.37500	2.23630	0.828
		Normal	-5.90000	2.10841	0.026***
	SPD	BPD	-1.37500	2.23630	0.828
		Normal	-7.27500	2.23630	0.008**
	normal	BPD	5.90000	2.10841	0.026***
		SPD	7.27500	2.23630	0.008**
**Total incorrect ** **responds**	BPD	SPD	-2.06250	1.67635	0.474
		Normal	4.45000	1.58047	0.025***
	SPD	BPD	2.06250	1.67635	0.474
		Normal	6.51250	1.67635	0.001*
	normal	BPD	-4.45000	1.58047	0.025***
		SPD	-6.51250	1.67635	0.001*

* P<0/001,

** P<0/01,

*** P<0/05

## Discussion

Differences were observed in facial emotion recognition and in neuropsychological function among patients with schizotypal personality disorder, borderline personality disorder, and normal population. The ability to interpret facial expression of emotion is necessary for social function during life, but it constantly changes through life (12). We concluded that participants in all 3 groups recognized happiness correctly. Happiness has been recognized as the most obvious emotion. The results revealed significant differences in the total correct and incorrect responses of emotion between BPD and normal group and SPD and normal group. There was a difference between BPD with normal group in fear responses and happiness responses. The result of this hypothesis is consistent with some studies (7, 10, (17), (18).

Linhan has argued that patients with BPD have high levels of vigilance and sensitivity when reacting to emotional signs. She found that borderline patients have more accurate perception compared with normal group. These patients are sensitive to internal or external resources of fear; they are oversensitive to fear (19). To confirm the finding, it can be said that patients with BPD may have the sense of rejection because of incorrect emotion recognition. Poor emotion recognition may cause a mechanism to have a poor emotional response and impaired interpersonal relationship in BPD patients.

Patients with BPD interpreted faces with fear emotion as unreliable and inaccessible ones. Although accurate perception of emotion is important for quality of life, these negative assessments of patients may lead to unstable close relationships and unbalanced quality of persistent relationship. Brain imaging studies have shown that amygdala responses increased toward negative emotions (20). There are differences between SPD and normal group in correct and incorrect responses to surprise emotion. There are not any consistent or inconsistent findings relating to this emotion in BPD patients. Choler et al. implied that patients with SPD have negative bias for vague faces, which may be due to social and interpersonal relationship deficits (21). Researches have mainly focused on negative emotions due to the role of negative emotion in personal problems of borderline personality patients (22). Moreover, differences have been observed in emotion recognition of disgust and anger in these patients.

Other findings revealed significant differences in correct recognition of disgust between BPD with SPD groups and SPD with normal groups. No studies compared these groups of patients. Patients with SPD perceived more faces as disgust emotion, which is consistent with Brown and Cohen’s findings. They demonstrated that patients with SPD misrecognize the neutral faces as disgust compared to the control group. This misrecognition may be related to their feelings of paranoia or social anxiety (11). The recent meta-analysis using facial expression stimuli found BPD patients have differences in disgust and anger emotion with normal group (23). However, this difference was not found in the present study; we only found an obvious evidence for brain location of disgust.

According to vulnerability stress approach, inappropriate social relations in patients with schizophrenia may be due to their disability in correctly recognize emotions, and leads to reduced social support. There is a relationship between schizophrenia and schizotypal personality disorder, so we can present the same conclusion for SPD. Patients with BPD have more rumination in routine communication. self-blame cause intense emotional reaction to negative stimuli and maladaptive behavior (24). These findings show incompatible and unstable social relationship about BPD. Two groups illustrated impaired recognition in disgust emotion, but the severity of this impairment is different between them. This difference may indicate the involvement of diverse brain regions. Separate pathology was indicated in facial emotional recognition in patients of both groups. The common feature of the patients with BPD and SPD is paranoid symptoms that may lead to negative perception of faces. Briefly, we conclude that disgust emotion has considerable importance in neurological perception of the 2 disorders. 

According to constant and maladaptive personality patterns in personality disorder, neuropsychological research could increase our concept of these disorders and realization of etiological factors. Because interpretation of emotion influences social communication, routine life, and thought, paying attention to patients’ deficits in this domain and discovering the differences will help accurate diagnosis of disorder. Considering the findings of this study, it can be stated that patients with BPD and SPD have deficits in emotion recognition. Brain magnetic resonance imaging studies have shown the role of amygdala in emotional circuits. Patients with BPD and SPD have demonstrated inappropriate functioning in emotion recognition and emotion processing speed, especially negative emotions such as disgust, anger, and fear. However, some studies did not find significant differences between the patients and normal group. These contradictory findings might be due to differences in personality disorder diagnosis, comorbidity with other personality disorders, or use of neurological instruments that were used in this study.

## Limitations

-Patients with medical problems and those with neurological deficits were ignored. 

-The participants were not matched for medications.

–The sample size was small.

Also, it is recommended that future research focus on the following topics: 

-Other neuropsychological functions should be investigated to better understand differences and similarities of the 2 disorders.

-Brain imaging methods should be used to examine brain regions involved in neurological assessment.

## Conclusion

Symptoms of the two disorders are associated with defect in neurological assessment. More attention to neurological aspect of these disorders can present a guideline in comorbidity assessment and treatment. Nevertheless, differences between the two disorders in the neurological domain of emotion were the main factors for diagnosis.

Considering the lack of studies in examining similarities and differences between the two disorders, it is necessary to conduct more studies in this area to achieve more reliable results.
